# Effects of Life Stage, Site, and Species on the Dragonfly Gut Microbiome

**DOI:** 10.3390/microorganisms8020183

**Published:** 2020-01-28

**Authors:** Sarah Nobles, Colin R. Jackson

**Affiliations:** Department of Biology, University of Mississippi, University, MS 38677, USA; smruss3913@gmail.com

**Keywords:** microbiome, dragonflies, insects, 16S rRNA, bacterial community, Odonata, bacterial diversity, nymph, adult

## Abstract

Insects that undergo metamorphosis from juveniles to adults provide an intriguing opportunity to examine the effects of life stage, species, and the environment on their gut microbiome. In this study, we surveyed the gut microbiomes of 13 species of dragonfly collected from five different locations subject to different levels of human impact. Juveniles were collected as nymphs from aquatic habitats while airborne adults were caught at the same locations. The gut microbiome was characterized by next generation sequencing of the bacterial 16S rRNA gene. Life stage was an important factor, with the gut microbiomes of dragonfly nymphs differing from those of adult dragonflies. Gut microbiomes of nymphs were influenced by sample site and, to a lesser extent, host species. Neither sample location nor host species had a strong effect on the gut microbiome of dragonfly adults. Regardless of life stage, gut microbiomes were dominated by members of the Proteobacteria, with members of the Bacteroidetes (especially in adults), Firmicutes, and Acidobacteria (especially in nymphs) also being proportionally abundant. These results demonstrate that different life stages of metamorphosing insects can harbor very different gut microbiomes and differ in how this microbiome is influenced by the surrounding environment.

## 1. Introduction

An animal’s microbiome plays a major role in the health and fitness of the host [1,2]. A healthy microbiome can increase host longevity and reproductive success, while an altered microbiome can increase the likelihood of disease and death. As DNA sequencing methods have become more accessible, microbiome analysis has moved beyond a focus on human health and into other areas of biology, including entomology [3]. The microbiomes of insects have been analyzed for a variety of reasons, including conservation and pest control [4]. More broadly, insects are among the most diverse and abundant animals on Earth and play key roles in many ecosystems [5]. Insects occupy a variety of habitats and the insect microbiome may, at least in part, depend on their surrounding environment [6]. The insect microbiome can also depend on its developmental stage, and insects vary in their lifecycle from holometabolous that completes a full cycle of metamorphosis from an egg to a larva to a pupa to an adult, to hemimetabolous that develops from an egg to a nymph to an adult, skipping the pupal stage [5]. Insects can show gut microbiome profiles that are specific to each developmental stage [6,7], and depending on the particular insect species, developmental stages can inhabit vastly different environments, for example being entirely aquatic as juvenile nymphs and entirely terrestrial as adults. The impacts of these major lifecycle changes on the gut microbiome have rarely been examined.

Dragonflies and damselflies (Order: Odonata) are hemimetabolous insects with a carnivorous juvenile nymph stage that can live in water for up to four years before emerging after a final molt into a terrestrial adult that can live for up to a year, feeding primarily on smaller flying insects. The few studies that have examined bacterial communities associated with dragonflies have focused on the gut microbiome of adults, typically using culture-dependent approaches [6,8,9,10]. These studies suggest that the gut microbiomes of adult dragonflies may be more diverse than that of other carnivorous insects [10], and that geographic location and season explain much of the variation in the composition of the adult dragonfly microbiome [9,10]. However, little is known of how the gut microbiome of dragonfly nymphs compares to that of adults, or how much the gut microbiome varies between dragonfly species.

Similarly, the extent to which human disturbance or development can influence the microbiomes of terrestrial or aquatic insects is unknown, even though habitat degradation is a major disturbance for aquatic insects [11]. Whether habitat-driven variation in the microbiome of aquatic nymphs would be carried into the adult stage is unclear, and no study has compared the gut microbiomes of nymphs and adults of the same dragonfly species. As hemimetabolous insects, dragonflies do not pass through the non-feeding pupal developmental stage that can result in substantial changes in the gut microbiome of holometabolous insects [12], and both dragonfly fly nymphs and adults are carnivorous, which could mitigate the effects that diet can have on the gut microbiome [13]. It is possible that the whole or part of the gut microbiome of nymphs could be retained in adult dragonflies, such that the aquatic environment in which a nymph develops could exert an influence on the adult microbiome. Dragonfly nymphs can be found in disturbed and pristine aquatic systems and are typically more tolerant of pollution and other human impacts than other aquatic insects [14]. Thus, dragonflies present an interesting system in which to explore the influence of host species, developmental stage, and the degree of human impact to the surrounding environment on the gut microbiome.

In this study we surveyed the gut microbiomes of 13 species of dragonfly nymphs and adults collected from five sites in Mississippi and Tennessee, USA. Sites varied in their degree of urbanization, and we hypothesized that dragonfly microbiomes would be more influenced by site than by host species or life stage. Our findings suggest that all three factors have a significant influence on the gut microbiome of dragonflies. 

## 2. Materials and Methods 

### 2.1. Sample Sites and Dragonfly Collection and Processing

Dragonflies were collected from five sites in north Mississippi and south Tennessee, USA. Sites were selected based on the current and historical land use patterns to reflect different levels of urbanization and potential human impact. The three sites in Mississippi were a vegetated pond with minimal human disturbance and no urbanization at the University of Mississippi Field Station (UMFS; 34°25′05.6″ N, 89°23′32.3″ W), a small lake at Camp Lake Stephens (CLS; 34°18′40.7″ N, 89°28′31.3″ W), a site used for summer youth camps but with only moderate ongoing human impacts, and a former treatment reservoir for a wastewater treatment plant that is close to a highway and agricultural land (Treatment Plant, TP; 34°16′36.7″ N, 89°31′01.4″ W). The two sites in Tennessee were in the city of Memphis; one was a lake downstream of horse barns in Shelby Farms Park (SF; 35°08′32.2″ N, 89°49′17.3″ W), a large urban park subject to much human activity, and the other was a drainage channel downstream of a large hospital within the Wolf River Greenway (WRG; 35°07′40.9″ N, 89°51′11.1″ W) that collects runoff from major roads and residential areas. Thus, sites ranged from WRG>SF>TP>CLS>UMFS in terms of potential human impact.

Dragonfly nymphs were collected from each site between January and April 2018 and adults were collected between May and June 2019. Ten individuals of each life stage were collected from each site to give a total of 50 nymphs and 50 adults. Adults were collected with nets while perching and nymphs were collected by dip netting into the dominant submerged material at each site (littoral vegetation at UMFS, CLS, and TP; leaf litter at WRG; and sediment at SF) Each collected individual was immediately placed in a sterile plastic bag and placed on ice for transportation back to the laboratory.

Dragonflies were measured for their length and weight and then washed in 70% ethanol prior to dissection. The gut tract was removed and vortexed in 1 mL sterile saline (0.8% NaCl) at maximum speed for 10 min. The resulting mixture was centrifuged (10,000× *g*, 15 min), the supernatant removed, and DNA extracted from the pellet.

### 2.2. DNA Extraction and Amplification

DNA was extracted using a DNeasy PowerSoil kit and protocol (Qiagen, Germantown, MD, USA). For microbiome analysis, DNA was amplified with barcoded primers targeting the V4 region of the bacterial 16S rRNA gene [15,16]. Amplification products were normalized using SequencePrep Normalization Plates (Life Technologies, Grand Island, NY, USA) and sequenced with an Illumina MiSeq platform at the Molecular and Genomics Core Facility at the University of Mississippi Medical Center. Extracted DNA was also used to identify dragonfly species. A portion of the CO1 gene was amplified using Odonata specific primers developed by Karthika et al. [17]. Forward primer OdoF1_t15′TGTAAAACGACGGCCAGTATTCAACHAATCATAARGATATTGG3′ and reverse primer OdoR1_t15′CAGGAAACAGCTATGACTAAACTTCTGGATGYCCRAARAAYCA3′ were used in reaction conditions described by Karthika et al. [17]. CO1 amplification products were sequenced through a commercial provider (Functional Biosciences, Madison, WI, USA). 

### 2.3. DNA Sequence Data Analysis

For 16S rRNA gene data, FASTQ files were processed using mothur version 1.40.5 following recommended procedures [15,18,19]. Sequences were aligned to the SILVA database (version 128) and classified according to the Ribosomal Database Project (RDP) database release 16. Sequences identified as unknown, mitochondria, or chloroplasts were removed from the dataset, as were sequences identified as potential chimeras. Sequences were clustered into operational taxonomic units (OTUs) defined by 97% sequence similarity. OTUs with just one or two sequence reads were removed prior to analyses of alpha and beta diversity, and diversity analyses were standardized by subsampling (1000 iterations) to the same number of sequence reads per sample (453 as defined by the number of remaining sequence reads in the lowest sample). Beta diversity was assessed using the abundance-based Bray–Curtis dissimilarity index and ordination through non-metric multidimensional scaling (NMDS). Analysis of similarity (ANOSIM) was used to determine if the composition of the gut microbiome was influenced by sample site, microhabitat, life stage, or species. Analysis of variance (ANOVA) in R version 3.0.2 was used to determine the influence of those variables on species richness (alpha diversity) on the gut microbiome.

For CO1 gene data, FASTA files were trimmed to retain confirmed bases and compared to those in GenBank (BLAST searches in January 2019) to determine dragonfly species identity. Species assignment was based on the top three BLAST results based on a BLAST “Ident” percentage of 96 or higher.

Sequence data is available in the NCBI Sequence Reads Archive under Bioproject PRJNA597338.

## 3. Results

### 3.1. Sampling and Sequencing Effectiveness

A total of 100 individual dragonflies were collected, however 13 (three nymphs and ten adults) yielded low numbers of 16S rRNA gene sequence reads and were excluded from the dataset. Of the remaining 87 individuals, CO1 gene sequencing identified them as belonging to 13 species, with eight species represented by both nymphs and adults (Table 1). Three adults showed poor CO1 sequencing and could not be assigned to a species. These adults were excluded from species-focused analyses but were retained for site- or life stage-focused analyses.

The number of dragonfly species collected varied by site and life stage and finding a nymph species at a given site did not relate to the later collection of adults (Table 1). Eight different species of adults were collected, and 13 different species of nymphs. One of the more rural sites (Camp Lake Stephens) had the highest number of species collected (eight) with *Erythemis simplicicollis* and *Celithemis elisa* being the most common with four individuals of each collected. The Shelby Farms and former Treatment Pond sites yielded the lowest number of dragonfly species (five). Across all sites, *E. simplicicollis* was the most collected species (19 individuals) followed by *Libellula luctuosa* (15 individuals) and *Pachydiplax longipennis* (13 individuals). Only one individual was collected for each *Erythrodiplax fusca*, *Ladonna deplanata*, and *Tetragoneuria cynosure* (Table 1).

### 3.2. Composition of the Dragonfly Gut Microbiome

**Figure 1:**
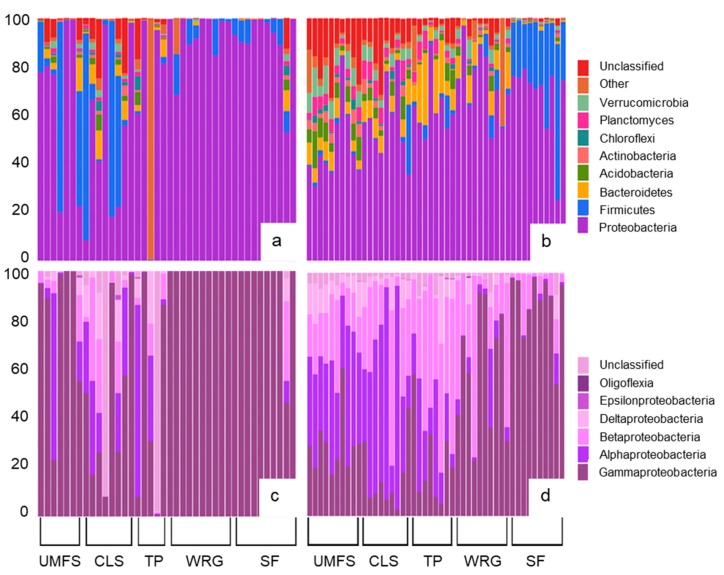
The composition of the gut microbiomes of dragonflies. (**a**) The percentage contribution of major bacterial phyla to the gut microbiome of dragonfly adults; (**b**) the percentage contribution of major bacterial phyla to the gut microbiome of dragonfly nymphs; (**c**) the percentage contribution of different subgroups of Proteobacteria to the Proteobacteria in dragonfly adults; and (**d**) the percentage contribution of different subgroups of Proteobacteria to the Proteobacteria in dragonfly nymphs. Each bar represents an individual dragonfly (from one of 13 species) collected from five sites in Mississippi and Tennessee, USA, along a gradient of potential human impact (WRG>SF>TP>CLS>UMFS).

The 87 dragonflies retained in the dataset yielded a total of 262,608 bacterial 16S rRNA gene sequences, at a mean of 3018 sequences per individual (range 503–27,053). Of these sequences, 13% could only be identified as unclassified Bacteria whereas 87% represented 33 different bacterial phyla. Four phyla (Proteobacteria, Firmicutes, Bacteroidetes, and Acidobacteria) accounted for 73% of all sequence recovered. Proteobacteria was most commonly the dominant phylum in both adults and nymphs, although the proportions of the different subphyla of Proteobacteria varied between the two life stages (Figure 1). Gut communities of adult dragonflies typically yielded more sequences identified as members of the Gammaproteobacteria than did those of nymphs, especially at the two sites (SF and WRG) more likely to be subject to human impacts, for which Gammaproteobacteria accounted for almost all of the Proteobacteria sequences detected (Figure 1). Nymphs at the SF site also had gut microbiomes dominated by Gammaproteobacteria, while nymphs at other sites typically had higher proportions of Alphaproteobacteria and Betaproteobacteria. The two life stages also showed some differences in the relative abundance of other bacterial phyla, with Firmicutes being the second most abundant phylum in adults and Bacteroidetes generally being the second most abundant phylum in nymphs (Figure 1). Verrucomicrobia, Planctomycetes, Chloroflexi, Acidobacteria, and Actinobacteria were all typically more prevalent in nymphs than adults, and nymphs tended to show more variation in the phyla comprising their gut microbiome (Figure 1).

16S rRNA gene sequences grouped into 8656 OTUs based on a 97% sequence similarity. Of these OTUs, 5571 were represented by just one or two sequence reads and were removed prior to analyses of community similarity and diversity, retaining 3085 OTUs. The four most prominent OTUs were identified as being members of the Gammaproteobacteria, three from family Enterobacteriaceae (accounting for a combined 60% of the sequences recovered from adult dragonflies and 21% from nymphs), and one from genus *Aeromonas* (accounting for 5% of the sequences from adults and 16% from nymphs; Table 2). Two of the OTUs (01 and 04) identified as members of the Enterobacteriaceae accounted for >50% of the sequences obtained from dragonfly adults but were much scarcer (2.4% of the sequences) in nymphs (Table 2). Other abundant OTUs were identified as belonging to the phylum Firmicutes (represented by the families Peptostreptococcaceae and Clostridiaceae, as well as the genus *Lactococcus*) and Fusobacteria (genus *Cetobacterium*). The sixth most abundant OTU identified as a member of the Chlamydiales (phylum Chlamydia), this OTU was largely found in one individual adult where it accounted for almost all of the sequences recovered (Table 2). Overall, the bacterial sequences recovered from dragonfly nymphs grouped into 2336 OTUs while the sequences recovered from adults grouped into 954 OTUs.

### 3.3. Variation in the Dragonfly Gut Microbiome by Life Stage, Species, and Environment

**Figure 2:**
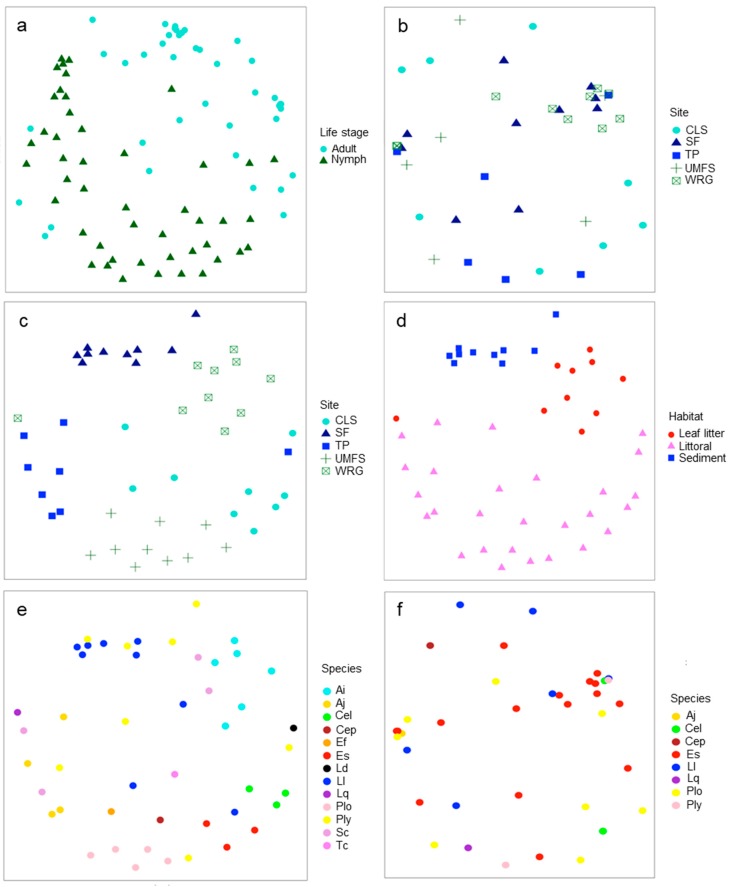
Non-metric multidimensional scaling (NMDS) ordinations of gut microbiomes of dragonflies grouped by life stage, site, habitat, or species. (**a**) Ordination by life stage (adults or nymphs, stress = 0.26); (**b**) ordination of adults by sample site (stress = 0.24); (**c**) ordination of nymphs by sample site (stress = 0.20); (**d**) ordination of nymphs by habitat samples (leaf litter, littoral vegetation, or sediment, stress = 0.24); (**e**) ordination of nymphs by dragonfly species (stress = 0.20); and (**f**) ordination of adults by dragonfly species (stress = 0.20). Sites represented a gradient of potential human impact (WRG>SF>TP>CLS>UMFS). Species were identified as *Anax imperator* (Ai), *Anax junius* (Aj), *Celithemis elisa* (Cel), *Celithemis eponina* (Cep), *Erythemis simplicicollis* (Es), *Erythrodiplax fusca* (Ef), *Ladona deplanata* (Ld), *Libellula luctuosa* (Ll), *Libellula quadrimaculata* (Lq), *Pachydiplax longipennis* (Plo), *Plathemix lydia* (Pl), *Sympetrum corruptum* (Sc), and *Tetragoneuria cynosure* (Tc).

In terms of the overall bacterial community composition, the dragonfly gut microbiome separated primarily by life stage with the microbiomes of nymphs and adults clearly separated in NMDS ordinations (Figure 2a; ANOSIM *p* < 0.001, R = 0.37). Treating each life stage separately, the gut microbiome of adults did not show clear effects of site (Figure 2b; ANOSIM *p* > 0.05), whereas the gut microbiome of nymphs showed a strong influence of site on its overall composition (Figure 2c; ANOSIM *p* < 0.001, R = 0.60). Part of the site effect on nymphs was likely driven by habitat differences between sites, as nymphs recovered from leaf litter, littoral vegetation at the edge of the site, or sediment could also be separated by the overall gut microbiome composition (Figure 2d; ANOSIM *p* < 0.001, R = 0.62). There was a suggestion that dragonfly host species influenced the overall composition of the microbiome in nymphs, but this was not quite significant (Figure 2e; ANOSIM *p* = 0.056). Host species was not significant in influencing the gut microbiome of dragonfly adults (Figure 2f; ANOSIM *p* > 0.05).

**Figure 3:**
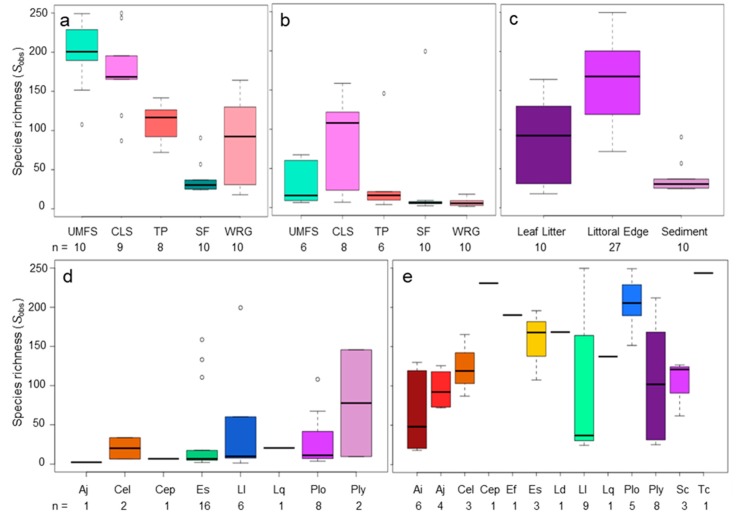
The observed species richness (*S*_obs_) in the gut microbiomes of dragonflies grouped by life stage and site, habitat, or species. *S*_obs_ is calculated as the number of operational taxonomic units detected when subsampling 453 16S rRNA gene sequences from each sample over 1000 iterations. *S*_obs_ is represented as boxplots with quartiles, including median line, outliers (circles), and whiskers representing the minimum and maximum *S*_obs_ for each sample type. (**a**) Dragonfly nymphs at sites representing a gradient of potential human impact (WRG>SF>TP>CLS>UMFS); (**b**) dragonfly adults at sites representing a gradient of potential human impact (WRG>SF>TP>CLS>UMFS); (**c**) dragonfly nymphs collected from different aquatic microhabitats, representing leaf litter, plants in the littoral edge, or sediment; (**d**) dragonfly adults separated by species; and (**e**) dragonfly nymphs separated by species. Species were identified as *Anax imperator* (Ai), *Anax junius* (Aj), *Celithemis elisa* (Cel), *Celithemis eponina* (Cep), *Erythemis simplicicollis* (Es), *Erythrodiplax fusca* (Ef), *Ladona deplanata* (Ld), *Libellula luctuosa* (Ll), *Libellula quadrimaculata* (Lq), *Pachydiplax longipennis* (Plo), *Plathemix lydia* (Ply), *Sympetrum corruptum* (Sc), and *Tetragoneuria cynosure* (Tc).

Alpha diversity of the dragonfly gut bacterial community was determined as the observed species richness (*S*_obs_) when subsampling the same number of sequences from each sample. *S*_obs_ varied with life stage, species, and site (ANOVA, *p* < 0.001 for all factors). Interactions between these variables were also significant, with life stage × site being the most significant (*p* = 0.0001), followed by species × site (*p* = 0.004), and life stage × species (*p* = 0.02). Dragonfly nymphs had a richer gut microbiome than adults at all five sites, although this richness varied between sites (Figure 3a,b). Nymphs at the two least impacted sites (UMFS and CLS) had more species rich gut microbiomes than nymphs at the other sites (*p* < 0.05; Figure 3a), while dragonfly adults showed no significant variation in the richness of their gut microbiome (Figure 3b). Gut microbiomes of nymphs collected from littoral vegetation at the edges of aquatic systems were also more species rich than those collected from leaf packs or sediment (*p* < 0.01; Figure 3c), likely reflecting the increased richness at sites UMFS and CLS (which were entirely littoral habitat).

Host dragonfly species effects on the richness of the gut microbiome were more complex (Figure 3d,e). Adults of *L. luctuosa* and *P. lydia* had richer gut microbiomes than the adults of other species, but this richness was highly variable (Figure 3d). The nymphs of these two species also tended to have richer gut communities than the nymphs of many other species, as did the nymphs of *P. longipennis* and *E. fusca* (Figure 3e). As implied by the significance of life stage on gut microbiome richness, the nymphs of any particular species tended to have richer gut bacterial communities than did the adults of the same species. Within individual species, *A. imperator* nymphs showed a strong negative correlation between body length and *S*_obs_ (r = 0.93, *p* < 0.01) and body mass and *S*_obs_ (r = −0.87, *p* < 0.05). In contrast, the nymphs of *E. simplicicollis* showed a positive correlation between mass and *S*_obs_ (r = 0.55, *p* < 0.05). No other species × life stage combinations had significant correlations between gut microbiome richness and body length or mass.

Since some dragonfly species were only sampled as adults or nymphs, there is the potential for effects of species and life stage to be confounded. Thus, we ran analyses on a reduced dataset containing only species for which both nymphs and adults were sampled, and for which >1 individual of each life stage × species combination was sampled (five species, *Celithemis elisa*, *Erythemis simplicicollis*, *Libellula luctuosa*, *Pachydiplax longipennis*, and *Plathemis lydia*, represented by 62 individuals). In terms of alpha diversity, this analysis confirmed the relationships between microbiome richness and life stage, species, and site seen in the full dataset, with significant relationships between *S*_obs_ and life stage (*p* < 0.0001), species (*p* < 0.01), and site (*p* < 0.001). As with the full dataset, interaction terms were also significant (life stage × site *p* < 0.0001; life stage × species *p* < 0.05; and species × site *p* < 0.01).

**Figure 4:**
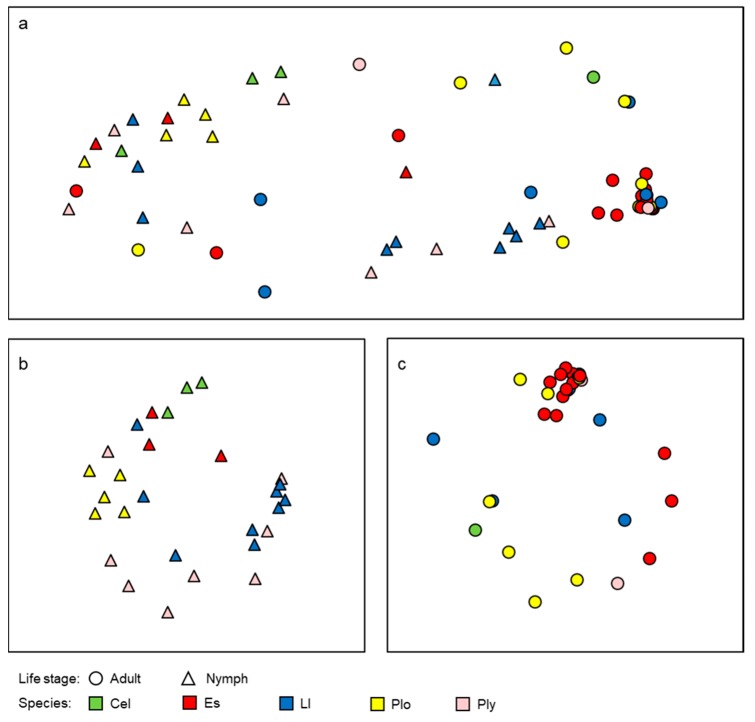
NMDS ordinations of gut microbiomes of five species of dragonflies grouped by life stage, or species. (**a**) Ordination by life stage (adults or nymphs, stress = 0.23); (**b**) ordination of nymphs by dragonfly species (stress = 0.24); and (**c**) ordination of adults by dragonfly species (stress = 0.19). Species were identified as *Celithemis elisa* (Cel), *Erythemis simplicicollis* (Es), *Libellula luctuosa* (Ll), *Pachydiplax longipennis* (Plo), and *Plathemix lydia* (Pl).

For patterns of beta diversity, this reduced dataset of five dragonfly species yielded similar patterns to the full dataset (Figure 4). Life stage was still an important factor separating the gut microbiomes of dragonflies, with nymphs having a distinct microbiome from adults (Figure 4a; ANOSIM *p* < 0.001, R = 0.34). Within each life stage, host species appeared to be more important in separating the gut microbiomes of nymphs (Figure 4b; ANOSIM *p* = 0.002, R = 0.26) than adults (Figure 4c; ANOSIM *p* = 0.045, R = 0.16), as was seen with the full dataset. Site also appeared to be a stronger factor influencing the gut microbiome of nymphs (ANOSIM *p* < 0.001, R = 0.72) than adults (ANOSIM *p* < 0.001, R = 0.32). However, the effects of site and species are more interlinked in this reduced dataset as with the smaller number of species sampled, there is increased potential for uneven distribution of species across the different sites (i.e., certain dragonfly species are more prevalent at some sites than others; Table 1).

## 4. Discussion

While the number of studies on insect microbiomes is increasing [20,21], few studies have compared the composition of the insect microbiome across life stages. This study is one of the first to investigate the gut microbial communities of dragonfly nymphs and adults, while also characterizing the variation in these gut communities across different environments and between host dragonfly species.

Dragonfly gut microbiomes were dominated by members of the Proteobacteria, consistent with the results of a prior study on adult dragonflies [9] and studies on other insects [22,23,24]. In a survey of the microbiomes of specimens representing eight different orders of insects (not including Odonata), Jones et al. [24] found that Gammaproteobacteria and Alphaproteobacteria were typically the most dominant subgroups of Proteobacteria, matching our findings for dragonfly adults and nymphs. While members of the Gammaproteobacteria typically dominated the gut microbiomes of adults, Alphaproteobacteria and Betaproteobacteria were more prevalent in dragonfly nymphs. Studies on the gut microbiomes of insect nymphs or larvae are limited, but Alphaproteobacteria and Betaproteobacteria have been found to be major components of the gut microbiome of nymphs of the European Firebug [25], the larvae of mosquitoes [7], and cockchafer beetles [26]. Dragonfly nymphs also tended to have higher proportions of Bacteroidetes in their gut microbial communities compared to adults, while adults typically had a higher proportion of Firmicutes, and similar patterns between adult and juvenile life stages have also been reported for beetles and ticks [26,27].

While comparisons at the phylum level provides some insights into patterns between life stages, finer scale examination in terms of specific OTUs provides more information on the potential roles of members of the dragonfly gut microbiome. Three of the four most abundant OTUs were identified as members of the Enterobacteriaceae (Gammaproteobacteria), a family of bacteria typically associated with enteric bacteria. However, the Enterobacteriaceae also includes the insect pathogen *Photorhabdus luminscens* and a variety of symbionts that can provide an insect host with a nutritional benefit or defense against colonization by pathogens [3,20,28,29]. Members of the Enterobacteriaceae have been detected previously in the guts of adult dragonflies [10], and while their potential role to the host is still unclear, their high prevalence and frequent occurrence suggests more than passive acquisition from the environment.

Another of the relatively abundant OTUs was identified as belonging to genus *Aeromonas* (Gammaproteobacteria), and this OTU was detected in 73% of the dragonfly samples. This genus was much less prevalent in a prior study of dragonfly gut communities [9], being found in only certain dragonfly species. That study and ours were in distant geographic areas (India and USA) and sampled very different dragonfly species, which could account for this discrepancy. The prior study also focused on culturable bacteria [9], and while *Aeromonas* can be cultured, its presence could have been masked by other more readily culturable taxa. *Aeromonas* includes species that can be symbiotic with an insect host, and a mutualistic relationship has been found between some species of *Aeromonas* and aquatic chironomid larvae, with the suggestion that the bacteria may protect the host from exposure to toxic metals [30]. *Wolbachia* (Alphaproteobacteria) have previously been reported as being prevalent in the dragonfly gut microbiome [10] but were barely detected in our study, accounting for less than 0.1% of all 16S rRNA gene sequences recovered, all from a single adult *Pachydiplax longipennis* host (where it accounted for almost 50% of the bacterial community). *Wolbachia* are insect parasites that alter reproductive behavior and physiology but may also provide protection against viral infections [31,32]. That they were limited to just one individual in this study suggests pathogen-like distribution in dragonflies with only infected hosts having these bacteria in their microbiome. The same could be said for an OTU identified as being within the phylum Chlamydia, which, while being fairly abundant when the entire dataset is looked at as a whole, was essentially confined to just a single adult dragonfly, where it accounted for almost all of the bacterial sequences recovered. This highlights the importance that adequate sample sizes are taken for microbiome studies, as a single infected host can dramatically distort our perception of the microbiome composition. This is especially important for wild-collected animals such as insects whose disease state may be difficult to determine visually.

Dragonfly gut microbiomes differed by life stage, a phenomenon that has been reported for other insects with life stages that occupy different habitats [7,26]. Juvenile insects have been found to have more species rich gut microbiomes than adults of the same species, consistent with our findings. To some extent this may reflect the surrounding environment as prior studies on juvenile insect microbiomes have examined the larvae of the mosquito *Anopheles gambiae* [7], which are aquatic and submerged in water, or the forest cockchafer, *Melolontha hipposatani* [26], whose larvae are buried and submerged in soil. Animals that are constantly immersed in a medium, whether water or soil, that contains a diverse inoculum of bacteria, could be expected to have a more diverse gut microbiome than those that are in less contact with their surroundings, because of the potential for greater colonization from the environment. Little to no work has been done on that area, but it could explain the reduced gut microbiome richness seen in some adult insects compared to larvae and nymphs.

The extent of human impact on the environment had a significant influence on the gut microbiome, especially for dragonfly nymphs. Nymphs collected from sites with the greatest level of human impact, the Shelby Farms site downstream from a horse farm, and the Wolf River Greenway downstream from a hospital, tended to have a less rich gut microbiome than nymphs from sites less likely to suffer disturbance. The gut microbial communities of aquatic organisms can shift when exposed to environmental contamination or pollution [33,34,35], and environmental disturbance was found to decrease the overall diversity of the oyster gut microbiome, primarily through the loss of rare phylotypes [36]. These studies and ours suggest that the gut microbiomes of invertebrates may be sensitive to anthropogenic disturbance of the environment, and hosts collected from sites subject to greater human impacts may harbor gut microbiomes that are less diverse than those from less impacted sites.

Finer scale environmental variation may have also had an influence on the gut microbiome, as nymphs collected from sediment had less rich gut microbiomes than those collected from vegetation at the littoral edge or from leaf packs. While habitat at the geographic scale can influence the insect gut microbiome [6], there has been no work examining how microhabitat variation can influence the structure of the insect gut microbiome. In the case of dragonfly nymphs, the nature of the microhabitat can influence the ability of the nymph to capture prey [37], so that differences between microhabitats could relate to dietary differences, which have previously been shown to influence the dragonfly gut microbiome [10]. For some insects, different juvenile instars inhabit different microhabitats [38] so that microhabitat differences could also relate to differences in the age of the host. Prior studies have found that the gut microbiomes of adult dragonflies differ by species [9,10], and we found that species was a significant influencer of the gut microbiome of both adults and nymphs. That said, no dragonfly species showed a gut microbial profile that was clearly distinct from that of other hosts. In part, the lack of clear separation between species was likely a result of the appreciable variation in gut microbiome composition within a species, even within individuals collected from the same sample location, and as a whole, the local environment appeared to be a stronger determinant of microbiome composition, particularly for nymphs.

The gut microbiome of insects is influenced by host diet [6,39,40] and seasonal variation in prey availability can be an important determinant of the gut microbiome of adult dragonflies [10]. Diet-driven seasonal changes have also been reported for the gut microbiome of mammals [41]. We did not assess the influence of seasonality or diet in this study, largely because nymphs and adults were sampled at different times of the year based on the organism’s life history. Each life stage was, however, collected within a particular time of year (winter/early spring for nymphs and spring/early summer for adults) so any seasonal affects within a life stage should be minimized. While seasonal patterns in prey availability may not have been important, differences in prey availability between sample sites may well have been. Habitat degradation from urbanization or human land use can result in changes in food availability, and this has been shown to affect the gut microbiome of mammals [42]. The significant effects of site on microbiome composition could be a reflection of differential prey availability at the more impacted sites. Prey availability is rarely considered when assessing spatial patterns in gut microbiomes between hosts collected from different locations but could be an explanation for variation in microbiome composition at geographic scales, as well as the variation between microhabitats.

This study is one of few to show how life stage is a major driver of the gut microbiome in insects, and we also found that site, especially in the context of potential impacts based on land use, exerts a strong influence on the microbiome of dragonfly nymphs and less of an effect on aerial adults. Adult dragonflies likely travel over broader ranges than nymphs, potentially lessening the effect of site, and nymphs are also continually exposed to the bacteria surrounding them based on the aquatic milieu they inhabit. Surprisingly, host species did not have as dramatic an effect on the gut microbiome of dragonflies as did life stage or site, but this could be a limitation of the experimental design, which was focused more on the latter two factors. The finding that dragonfly nymphs and adults have substantially different gut microbiomes does bring up questions as to what really is the microbiome of a host that has substantially different life stages. For mammals, there is a tendency to view the adult as having the mature microbiome [43], but in insects such as dragonflies, the adult form may be relatively short-lived compared to the nymph. While the same species, and even the same individual, a dragonfly nymph and the adult it becomes are essentially different holobionts. They inhabit entirely different environments, prey on different food, and, as shown here, have fundamental differences in their gut microbiome and how it is influenced by habitat variability.

## Figures and Tables

**Table 1:** Dragonflies collected for the analysis of their gut microbiome from five sites ranging from low to high potential human impact (the University of Mississippi Field Station (UMFS), Camp Lake Stephens (CLS), a disused wastewater treatment plant (TP), Wolf River Greenway (WRG), and Shelby Farms Park (SF)). Dragonfly adults were collected while perching while nymphs were collected from submerged littoral vegetation, sediment, or leaf litter.

Sample ID	Life Stage	Mass (g)	Length (mm)	Site	Habitat	Species ^1^
A01	Adult	0.22	46	WRG	Perch	*Libelllula luctuosa*
A02	Adult	0.30	46	WRG	Perch	*Erythemis simplicicollis*
A03	Adult	0.22	49	WRG	Perch	*Erythemis simplicicollis*
A04	Adult	0.06	25	WRG	Perch	*Libelllula luctuosa*
A05	Adult	0.22	45	WRG	Perch	*Erythemis simplicicollis*
A06	Adult	0.22	47	WRG	Perch	*Erythemis simplicicollis*
A07	Adult	0.26	48	WRG	Perch	*Pachydiplax longipennis*
A08	Adult	0.23	45	WRG	Perch	*Erythemis simplicicollis*
A09	Adult	0.30	45	WRG	Perch	*Pachydiplax longipennis*
A10	Adult	0.21	44	WRG	Perch	*Erythemis simplicicollis*
A11	Adult	0.23	45	SF	Perch	*Anax junius*
A12	Adult	0.22	47	SF	Perch	*Erythemis simplicicollis*
A13	Adult	0.11	42	SF	Perch	*Pachydiplax longipennis*
A14	Adult	0.22	45	SF	Perch	*Erythemis simplicicollis*
A15	Adult	0.24	48	SF	Perch	Unknown
A16	Adult	0.26	42	SF	Perch	*Erythemis simplicicollis*
A17	Adult	0.30	46	SF	Perch	*Erythemis simplicicollis*
A18	Adult	0.50	45	SF	Perch	*Erythemis simplicicollis*
A19	Adult	0.19	44	SF	Perch	*Libelllula luctuosa*
A20	Adult	0.24	44	SF	Perch	*Erythemis simplicicollis*
A21	Adult	0.40	50	CLS	Perch	*Erythemis simplicicollis*
A23	Adult	0.41	50	CLS	Perch	*Celithemis elisa*
A24	Adult	0.21	46	CLS	Perch	Unknown
A25	Adult	0.25	45	CLS	Perch	*Erythemis simplicicollis*
A26	Adult	0.31	49	CLS	Perch	*Orthetrum glaucum*
A28	Adult	0.17	40	CLS	Perch	*Libelllula luctuosa*
A29	Adult	0.05	23	CLS	Perch	*Pachydiplax longipennis*
A30	Adult	0.20	40	CLS	Perch	*Erythemis simplicicollis*
A32	Adult	0.18	40	TP	Perch	*Pachydiplax longipennis*
A35	Adult	0.14	36	TP	Perch	*Plathemis lydia*
A36	Adult	0.29	43	TP	Perch	*Plathemis lydia*
A37	Adult	0.20	40	TP	Perch	*Pachydiplax longipennis*
A39	Adult	0.24	41	TP	Perch	*Libellula quadrimaculata*
A40	Adult	0.24	45	TP	Perch	Unknown
A41	Adult	0.29	48	UMFS	Perch	*Erythemis simplicicollis*
A44	Adult	0.17	44	UMFS	Perch	*Libelllula luctuosa*
A45	Adult	0.20	39	UMFS	Perch	*Pachydiplax longipennis*
A48	Adult	0.23	44	UMFS	Perch	*Pachydiplax longipennis*
A49	Adult	0.18	33	UMFS	Perch	*Celithemis elisa*
A50	Adult	0.17	41	UMFS	Perch	*Libelllula luctuosa*
N01	Nymph	0.06	13	UMFS	Littoral	*Plathemis lydia*
N02	Nymph	0.07	12	UMFS	Littoral	*Pachydiplax longipennis*
N03	Nymph	0.04	10	UMFS	Littoral	*Pachydiplax longipennis*
N04	Nymph	0.05	10	UMFS	Littoral	*Pachydiplax longipennis*
N05	Nymph	0.07	12	UMFS	Littoral	*Pachydiplax longipennis*
N06	Nymph	0.03	8	UMFS	Littoral	*Erythrodiplax fusca*
N07	Nymph	0.11	10	UMFS	Littoral	*Erythemis simplicicollis*
N08	Nymph	0.12	11	UMFS	Littoral	*Erythemis simplicicollis*
N09	Nymph	0.06	9	UMFS	Littoral	*Celithemis eponina*
N10	Nymph	0.04	10	UMFS	Littoral	*Pachydiplax longipennis*
N11	Nymph	0.24	17	CLS	Littoral	*Tetragoneuria cynosura*
N13	Nymph	0.06	9	CLS	Littoral	*Libelllula luctuosa*
N14	Nymph	0.03	7	CLS	Littoral	*Erythemis simplicicollis*
N15	Nymph	0.10	10	CLS	Littoral	*Celithemis elisa*
N16	Nymph	0.07	11	CLS	Littoral	*Celithemis elisa*
N17	Nymph	0.24	20	CLS	Littoral	*Ladonna deplanata*
N18	Nymph	0.10	13	CLS	Littoral	*Celithemis elisa*
N19	Nymph	0.05	11	CLS	Littoral	*Libelllula luctuosa*
N20	Nymph	0.01	6	CLS	Littoral	*Plathemis lydia*
N21	Nymph	0.22	26	TP	Littoral	*Anax junius*
N22	Nymph	1.71	50	TP	Littoral	*Anax junius*
N23	Nymph	0.36	22	TP	Littoral	*Sympetrum corruptum*
N24	Nymph	0.30	30	TP	Littoral	*Anax junius*
N26	Nymph	1.49	47	TP	Littoral	*Anax junius*
N27	Nymph	0.45	25	TP	Littoral	*Plathemis lydia*
N28	Nymph	0.23	20	TP	Littoral	*Sympetrum corruptum*
N30	Nymph	0.09	13	TP	Littoral	*Plathemis lydia*
N31	Nymph	0.22	24	WRG	Leaves	*Anax imperator*
N32	Nymph	1.32	43	WRG	Leaves	*Anax imperator*
N33	Nymph	0.53	23	WRG	Leaves	*Libelllula luctuosa*
N34	Nymph	0.66	36	WRG	Leaves	*Anax imperator*
N35	Nymph	1.09	10	WRG	Leaves	*Anax imperator*
N36	Nymph	0.01	9	WRG	Leaves	*Sympetrum corruptum*
N37	Nymph	1.67	47	WRG	Leaves	*Neodythemis preussi*
N38	Nymph	0.06	9	WRG	Leaves	*Sympetrum obtrusum*
N39	Nymph	1.35	44	WRG	Leaves	*Anax imperator*
N40	Nymph	0.19	24	WRG	Leaves	*Anax imperator*
N41	Nymph	0.15	20	SF	Sediment	*Plathemis lydia*
N42	Nymph	0.11	15	SF	Sediment	*Plathemis lydia*
N43	Nymph	0.32	20	SF	Sediment	*Libelllula luctuosa*
N44	Nymph	0.22	17	SF	Sediment	*Libelllula luctuosa*
N45	Nymph	0.14	20	SF	Sediment	*Libelllula luctuosa*
N46	Nymph	0.43	21	SF	Sediment	*Libelllula luctuosa*
N47	Nymph	0.33	25	SF	Sediment	*Libelllula luctuosa*
N48	Nymph	0.29	19	SF	Sediment	*Plathemis lydia*
N49	Nymph	0.07	13	SF	Sediment	*Plathemis lydia*
N50	Nymph	0.44	25	SF	Sediment	*Libelllula luctuosa*

^1^ As determined from partial CO1 gene sequencing.

**Table 2:** The 20 most abundant operational taxonomic units (OTUs) of bacteria identified in the gut microbiome of dragonfly adults and nymphs collected from five sites in Mississippi and Tennessee, USA. Thirteen different species of dragonflies were included in the dataset.

OTU	Number of Sequences	% Total Sequences	% Adult Sequences	% Nymph Sequences	Finest Classification (Phylum)
01	35,565	13.9	31.7	2.1	Enterobacteriaceae (Proteobacteria)
02	33,718	13.2	3.9	19.3	Enterobacteriaceae (Proteobacteria)
03	30,561	12.0	5.2	15.8	*Aeromonas* (Proteobacteria)
04	24,585	9.6	24.4	0.3	Enterobacteriaceae (Proteobacteria)
05	9472	3.7	0.2	6.1	Peptostreptococcaceae (Firmicutes)
06	8636	3.4	9.0	0.4	Chlamydiales (Chlamydia) ^1^
07	6710	2.6	0.1	4.1	*Cetobacterium* (Fusobacteria)
08	5790	2.3	0.0	3.9	Comamonadaceae (Proteobacteria)
09	5488	2.1	3.3	1.2	*Lactococcus* (Firmicutes)
10	4179	1.6	0.0	3.7	Clostridiaceae (Firmicutes)
11	3640	1.4	0.0	2.4	*Novosphingobium* (Proteobacteria)
12	3088	1.2	0.0	1.9	Alphaproteobacteria (Proteobacteria)
13	2962	1.2	0.0	1.9	Clostridiaceae (Firmicutes)
14	2464	1.0	0.0	1.6	Burkholderiales (Proteobacteria)
15	2411	0.9	0.0	1.6	Proteobacteria
16	2037	0.8	0.0	1.3	Acinetobacter (Proteobacteria)
17	1847	0.7	0.0	1.2	Bacillales (Firmicutes)
18	1626	0.6	0.0	1.1	Enterobacteriaceae (Proteobacteria)
19	1441	0.5	1.3	1.1	Lactococcus (Firmicutes)
20	1384	0.5	0.0	0.9	Neisseriaceae (Proteobacteria)

^1^ A single adult dragonfly accounted for 99% of the sequences assigned to OTU06.
